# A patient with chronic lymphocytic leukemia and acquired angioedema: correlation of clinical and biochemical response to CLL therapy

**DOI:** 10.3332/ecancer.2013.292

**Published:** 2013-02-14

**Authors:** GR Mohyuddin, I Rabinowitz

**Affiliations:** University of New Mexico Cancer Center, 1201 Camino de Salud NE, Albuquerque, NM 87131, USA

## Abstract

Acquired angioedema (AAE) is a result of an acquired deficiency or inactivity of the C1 esterase inhibitor (C1-INH). There is a well-known link between AAE and lymphoplasmacytic disorders.

A 65-year-old woman who was diagnosed with chronic lymphocytic leukemia (CLL), presented with recurrent episodes of angioedema. Although no association between the CLL and angioedema was initially recognized, further workup showed her to have low C1-INH levels. Chemotherapy helped prevent subsequent episodes, but three years later she redeveloped angioedema. She was then placed on ofatumumab maintenance and has since remained free of angioedema.

Knowledge of this rare disease and anticipation of the link between CLL and AAE can prevent further attacks and associated morbidity.

## Introduction

AAE is a result of an acquired deficiency or inactivity of C1-INH. The excess complement and bradykinin activity that ensues causes the clinical manifestations of angioedema, such as laryngeal edema, skin edema, and abdominal pain [1, 2].

There is a well known link between AAE and lymphoplasmacytic disorders [1, 3]. Recognition and understanding of the disease associations may result in treatments that may help prevent complications and recurrences, as our case highlights.

## Case description

In November 2007, a 65-year-old woman presented to our oncology clinic, after an acute event of angioedema, that required intubation and hospitalization in the ICU for two weeks. She had been diagnosed with CLL in July 2007, after presenting with splenomegaly and lymphocytosis. She had not been started on therapy, as she was asymptomatic. Her co-morbidities included osteoarthritis and hypertension. The medications she was regularly taking at that time included calcium, vitamin D, and hydrochlorothiazide. She had no previous history of atopy or angioedema.

The patient described the episodes beginning as a tingling around her face and mouth similar to a bee sting, swelling of her mouth and finally, a complete inability to breathe. There were no inciting episodes that the patient could point to that caused this to occur.

In April 2008, she again developed an attack of angioedema, necessitating another intubation and hospitalization for a few days. This time a complete workup to evaluate her angioedema was done. During her second episode, her C1 esterase level was 4 mg/dL (reference value, >11 mg/dL) and her C1 esterase activity was 5% (reference range, 68%–200%). Her rheumatoid factor was elevated at 87. Her C1Q binding assay and complement 3 levels were normal, but complement 4 levels were noticeably low. Interestingly, when we repeated these studies one month later, her C1 esterase activity had risen to 53%, which may in part have been due to a tapering course of steroids that she received after her second episode.

Given the association between acquired angioedema and CLL, we decided to initiate chemotherapy, and repeat the C1 esterase levels after completion of six cycles of rituximab, cyclophosphamide, vincristine and prednisone (R-CVP). Her chemotherapy was complicated by a severe reaction to rituximab during the second cycle and it was subsequently discontinued from the regimen. She completed six cycles of cyclophosphamide, vincristine and prednisone (CVP) in October 2008. At the end of chemotherapy, her C1 esterase level and function were 12 mg/dL and 55%, respectively. She was referred to rheumatology, who offered her therapy with hydroxycholoroquine, but she refused. Our patient thus received no further treatment for her angioedema. Our patient remained stable until August 2011, when she had a repeat episode of angioedema. She was not intubated this time; however, she received corticosteroids, fresh frozen plasma and diphenhydramine. Her last C1 esterase function was 12% in May 2011.

At that time we did not offer her any further treatment for her CLL, which showed no evidence of relapse on blood tests and CT scans. Her lymphocytosis and splenomegaly had resolved and remained normal.

Within two months in October 2011, she had another recurrence of angioedema, which again was treated with corticosteroids, fresh frozen plasma and diphenhydramine. Her C1-INH function in December 2011 was 25%. In reviewing the patient’s history, we noted that although her C1-INH levels failed to normalize after chemotherapy, clinically she had no episodes of angioedema for three years thereafter. As the patient previously was allergic to rituximab, we decided to try ofatumumab. The patient was treated with ofatumumab beginning November 2011, receiving seven weekly doses and then on a monthly schedule for another four doses. As of January 2013, the patient has remained free from any recurrences of angioedema for over a year of follow-up.

## Discussion

Autoimmune complications can be seen in 25% of CLL patients. Most common is autoimmune haemolytic anaemia. Immune thrombocytopenia, pure red cell aplasia, and autoimmune neutropenia can also be seen although less common, whereas nonhaematological autoimmunity such as angioedema is rare [4].

AAE is in itself an extremely rare entity, with around 150 cases reported [3]. It is broadly classified in two types: Type I is characterized by both low levels and function of C1-INH in the circulation and the Type II form is associated with normal levels of C1-INH, but a low function of C1-INH [5].

Although no uniform criteria for diagnosis exists, its diagnostic features include low C1-INH activity, very low C4 levels and low C3 levels. Autoantibodies to C1-INH can be shown in up to 70% of patients with AAE. The aforementioned features, in addition to a classic clinical picture and absence of other causative factors, can lead one to a diagnosis [1].

Abnormalities in this condition include an increased consumption of C1-INH and excessive activation of the classical complement pathway. Due to low levels of C1-INH, C1Q and C4, angioedema symptoms keep recurring and presenting with edema to the upper respiratory tract, the gastrointestinal tract and tongue. AAE typically presents in the fourth decade of life, or later, in contrast to the hereditary form [6].

To the best of our knowledge, there were 17 previous cases of CLL and small lymphocytic lymphoma associated with angioedema [3, 4, 7–9]. However, there exists abundant literature on the association of AAE with lymphoplasmacytic disorder of different types. While reviewing 32 patients with AAE, Castelli discovered that 13 (40%) had MGUS and nine (28%) had lymphoproliferative disease [3].

The Food and Drug Administration has approved the C1-INH concentrate, Berinert P, and a kallikrein inhibitor ecallantide, Kalbitor, for the treatment of acute attacks. In cases where these are not available such as in our patient, fresh frozen plasma can be given [10].

Icatiban is a bradykinin B2 receptor antagonist, which is an established therapy for hereditary angioedema. In a recent study, it was also shown to have success in treating acute attacks of acquired angioedema. Eight patients were studied, and of a total of 48 attacks, 47 achieved complete resolution with a single subcutaneous injection [11].

Long-term treatment involves addressing the underlying condition, or using antifibrinolytic drugs or danazol, an agent that increases C1-INH synthesis and has been shown to be efficacious [12].

**Figure 1:**
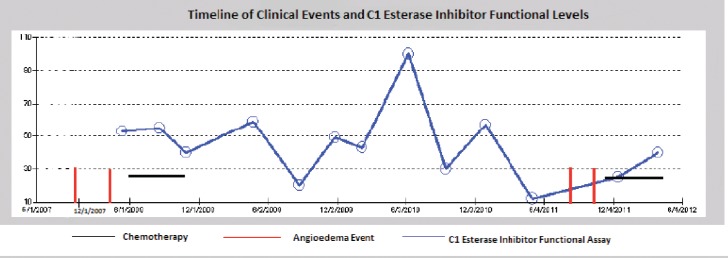
Time line of episodes of angioedema correlated to treatment of CLL. Anti-CLL therapy has afforded the patient significant time without angioedema attacks. The C1 esterase inhibitor functional assay shows a partial correlation between the level and the occurrence of the angioedema attacks.

As noted in Figure 1, there seems to be a relationship between treatment of the underlying CLL and the frequency of angioedema attacks. The patient had episodes of angioedema in early 2008 when her C1 esterase was low (5%) and in late 2011 when her C1 esterase was also low again (12% in May and 25% in December). However, there were times when the patients C1-INH was low and she had no episode of angioedema (20% in August 2009). Although there seems to be a relationship between the C1 esterase functional assay and her angioedema events, it is not very strongly correlated. Her CLL was in remission since her first chemotherapy in 2008 with no recurrence of splenomegaly or lymphocytosis. Thus the angioedema does not seem to be correlated with her CLL disease status. It could be that the treatments used for CLL, produce a sufficient immunosuppressive state to prevent further angioedema attacks, independent of its effect on the tumor. A part of that immunosuppression is reflected in the rise of the C1 esterase functional assay level, but it is certainly possible that other aspects of the immune system that are not tested by our assays play a role in recurrent attacks.

## Conclusion

The patient presents yet another example of the association between lymphoproliferative disorders and AAE. However, the patient also displays the complexity of this association. After careful review of the data, it is unclear whether the angioedema attacks are related to the lymphoproliferative disease status, the quantitative C1 esterase functional assay, some other factors that were not tested, or a combination of these and other variables.

## Conflicts of Interest

The authors have no conflicts of interest to declare.
